# Editorial: Current insights in Epigenetics and Epigenomics

**DOI:** 10.3389/freae.2025.1566501

**Published:** 2025-02-07

**Authors:** Steven Henikoff, Sharon Y. R. Dent, Raul Mostoslavsky, Ting Wang, Luca Comai

**Affiliations:** ^1^ Basic Sciences Division, Fred Hutchinson Cancer Center, Seattle, WA, United States; ^2^ Howard Hughes Medical Institute, Chevy Chase, MD, United States; ^3^ Department of Epigenetics and Molecular Carcinogenesis and Center for Cancer Epigenetics, University of Texas MD Anderson Cancer Center, Houston, TX, United States; ^4^ University of Texas MD Anderson Cancer Center/UT Health Houston Graduate School of Biomedical Sciences, Houston, TX, United States; ^5^ The Krantz Family Center for Cancer Research, The Massachusetts General Hospital Cancer Center and Harvard Medical School, Boston, MA, United States; ^6^ The Broad Institute of Harvard and MIT, Cambridge, MA, United States; ^7^ Department of Genetics, Washington University in St. Louis, St. Louis, MO, United States; ^8^ Department of Plant Biology, University of California, Davis, Davis, CA, United States

**Keywords:** Epigenetics, Epigenomics, DNA methylation, chromatin, environment

We are starting this new year with our first full collection of selected papers, consisting of review and research articles that provide glimpses into some of the exciting current areas of research in the broad field of epigenetics and epigenomics. Below we highlight topics reviewed in this collection with examples of new research published in Frontiers in Epigenetics and Epigenomics during 2024.

No scientific topic has been more in the news than climate change and the increasing frequency of disasters that have resulted (Roe et al.). What might be the epigenetic and epigenomic consequences of disastrous events? An especially well-studied disaster resulting in epigenetic modifications was the Dutch Hunger Winter, the 1944-45 famine during the Nazi occupation of Holland, highlighted in a review article in this series (Bernasocchi and Mostoslavsky). Physiological and behavioral effects were observed in children exposed to starvation *in utero*, and the genetic and epigenetic mechanisms underlying the predisposition of chronic conditions such as obesity and diabetes is an expanding area of research.

Disasters such as earthquakes and droughts can have mental health effects and associated epigenetic modifications (Roe et al.). Nearly all of the epigenetic studies examined have measured DNA methylation, although plausible mechanisms for environmentally induced changes in DNA methylation affecting human behavior are lacking (Bird, 2024). Even in plants, where meiotically heritable and environmentally induced DNA methylation epialleles are well-documented, evidence for their evolutionary importance is inconclusive (Comai, 2023). Other epigenetic modifiers, especially small RNAs, have received relatively little attention despite their demonstrated importance in regulating physiological processes. For example, microRNAs are known to regulate differentiation by targeting specific sets of mRNAs for degradation (Razavi-Mohseni and Beer, 2024). There are thousands of microRNA genes in the human genome, and we expect to see more environmental studies focused on microRNAs, which won the 2024 Nobel Prize in Physiology and Medicine more than 3 decades after their discovery.

Environmental changes that affect diet can have metabolic impacts on synthesis of the universal methyl donor, S-adenosyl methionine, which in turn affects not only DNA methylation, but also histone methylation, modifications that condition the epigenomic landscape (Bernasocchi and Mostoslavsky). Vitamins and other dietary micronutrients can alter the activities of the DNMT family of DNA methyltransferases and the TET family of enzymes that oxidize 5-methylcytosine to modify and remove the DNA methyl mark (Leesang et al.). DNA methylation and silencing histone modifications may be inherited through multiple rounds of cell division, and changes in both modifications have important implications for our understanding of cancer and aging.

Although most attention in the chromatin field has been on histone modifications, histone variants are critical for maintaining the chromatin landscape, especially post-mitotically, when canonical S-phase-dependent histones are silent. In addition, specialized histone variants are critical for fundamental cellular processes, including chromosome segregation (CENP-A), transcriptional regulation (H2A.Z) and DNA repair (H2A.X). DNA double-strand breaks have long been known to trigger rapid phosphorylation of H2A.X, but what happens next depends on the genomic and epigenomic context of the break (Clerf et al.). Multiple repair pathways for homologous recombination and non-homologous end-joining require nucleosomes to be destabilized for the DNA repair machineries to gain access, and this is facilitated by increases in H2A.Z acetylation and incorporation of macroH2A.

The chromatin dynamics required to make DNA available for replication, transcription and repair are mediated by four major families of chromatin remodelers, which share structurally related ATP-dependent DNA translocase domains. Subunits of the SWI/SNF family of remodelers, which acts to clear enhancers and promoters of nucleosomes to facilitate engagement of RNA Polymerase II, and possibly histone acetylation as well (Zhang, 2024), are mutated in >20% of human cancers. Just how SWI/SNF complexes interact with transcription factors, such as the androgen receptor (AR) in prostate cancer, has been the subject of much recent research (Ordonez-Rubiano et al.). Transcription factors (TFs), such as AR, provide sequence and cell-type specificity for the action of chromatin regulatory complexes and modifications. Similarly, a group of TFs collectively known as Phytochrome Interacting Factors (PIFs) provide sequence specificity for “landscaping” the epigenome in plants (Ammari et al.), acting as recruiters of chromatin regulators. Networks of oncogenic transcription factors are the major drivers of cancer, either as oncogenes when up-regulated or as tumor suppressors when down-regulated. In one study in this series, the authors combined RNA sequencing and ATAC-seq with CUT&Tag chromatin profiling for active and silencing histone modifications to follow the time-course of epigenomic alterations after oncogene induction (Vasilopoulos and Martinez-Zamudio). Detailed maps of chromatin changes following induction may help to guide the development of therapies to favor senescence over uncontrolled proliferation.

Among the most difficult cancers to treat are Ewing’s sarcomas (EWS), the subject of a clinical epigenomics study in this series (Patrizi et al., 2024). Deconvolution of DNA methylation bead-chip data from 32 EWS pediatric patients quantified the total numbers and percentages of immune cells in the tumors without requiring a tumor-specific signature. As the degree of immune infiltration correlates with longer survival, DNA methylation profiling may serve as a general cancer diagnostic. DNA methylation levels measured in peripheral blood can also be used as a disease diagnostic: As was reported in another clinical study in this series, the level of blood DNA methylation at the promoter of the corticotropin-releasing factor gene in Cushing’s Syndrome patients correlated with amygdala volume and depression relative to healthy controls (Lee et al.). We look forward to further technological improvements in DNA methylation profiling (Deinichenko et al., 2024; Xu et al., 2024) and other epigenomic interrogation techniques, not only for disease diagnosis, but also for understanding fundamental principles of genetics and cell biology that epigenomic research can reveal.
